# Case Report: Overlapping Multiple Sclerosis With Anti-N-Methyl-D-Aspartate Receptor Encephalitis: A Case Report and Review of Literature

**DOI:** 10.3389/fimmu.2020.595417

**Published:** 2020-12-09

**Authors:** Ying Huang, Qian Wang, Silin Zeng, Yaqing Zhang, Liangyu Zou, Xuejun Fu, Qianhui Xu

**Affiliations:** Department of Neurology, Shenzhen People’s Hospital (The Second Clinical Medical College, Jinan University, The First Affiliated Hospital, Southern University of Science and Technology), Shenzhen, China

**Keywords:** anti-NMDA receptor encephalitis, multiple sclerosis, atypical symptoms, overlap, anti-NMDA antibody

## Abstract

Anti-N-methyl-D-aspartate receptor (NMDAR) encephalitis is an autoimmune disorder mediated by NMDAR antibodies, typically manifesting as behavioral complaints, psychosis, seizures, movement disorders, hypoventilation, and autonomic dysfunction. In recent years, the predisposing factors and pathophysiological mechanisms of anti-NMDAR encephalitis have been tried to be clarified. It has been recognized that an overlap may be observed between anti-NMDAR encephalitis and inflammatory demyelinating disease. However, anti-NMDAR encephalitis is rarely associated with multiple sclerosis. Here, we describe a Chinese female patient diagnosed with relapsing remitting multiple sclerosis who developed anti-NMDAR encephalitis. Further, we discuss the previously reported literature.

## Introduction

Anti-N-methyl-D-aspartate receptor (NMDAR) encephalitis is an autoimmune disorder that is mediated by NMDAR antibodies. It is characterized by the manifestation of behavioral complaints, psychosis, seizures, movement disorders, hypoventilation, and autonomic dysfunction (1). It has been recognized that an overlap may be observed between anti-NMDAR encephalitis and inflammatory demyelinating disease, particularly with neuromyelitis optical spectrum disorder, myelin oligodendrocyte glycoprotein associated disorders, and acute demyelinating encephalomyelitis (2). However, it is seldom associated with multiple sclerosis (MS). Here, we report a female patient diagnosed with relapsing remitting multiple sclerosis (RRMS) who developed anti-NMDA-R encephalitis. To the best of our knowledge, the overlapping of the two diseases has not been reported previously in Chinese patients.

## Case Description

**Figure 1:**
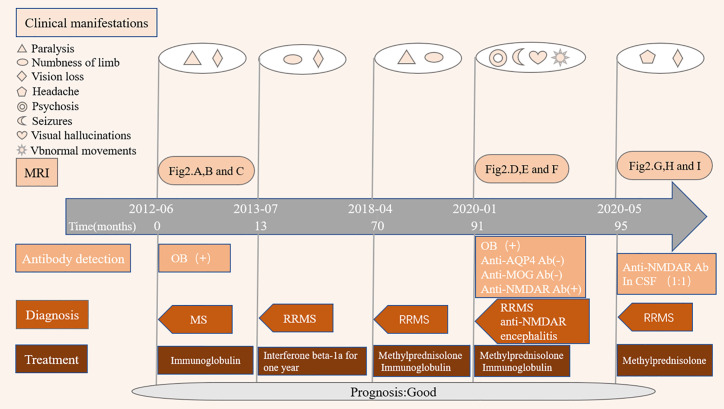
Disease course of the patient. MRI, Magnetic resonance imaging; OB, oligoclonal immunoglobulin bands; MS, multiple sclerosis; RRMS, relapsing remitting multiple sclerosis; Anti-NMDAR encephalitis, Anti-N-methyl-D-aspartate receptor encephalitis; Anti-AQP4 ab, Anti-aquaporin 4 antibody; Anti-MOG ab, Anti-myelin oligodendrocyte glycoprotein antibody; anti-NMDAR ab, Anti-N-methyl-D-aspartate receptor antibody.

**Figure 2:**
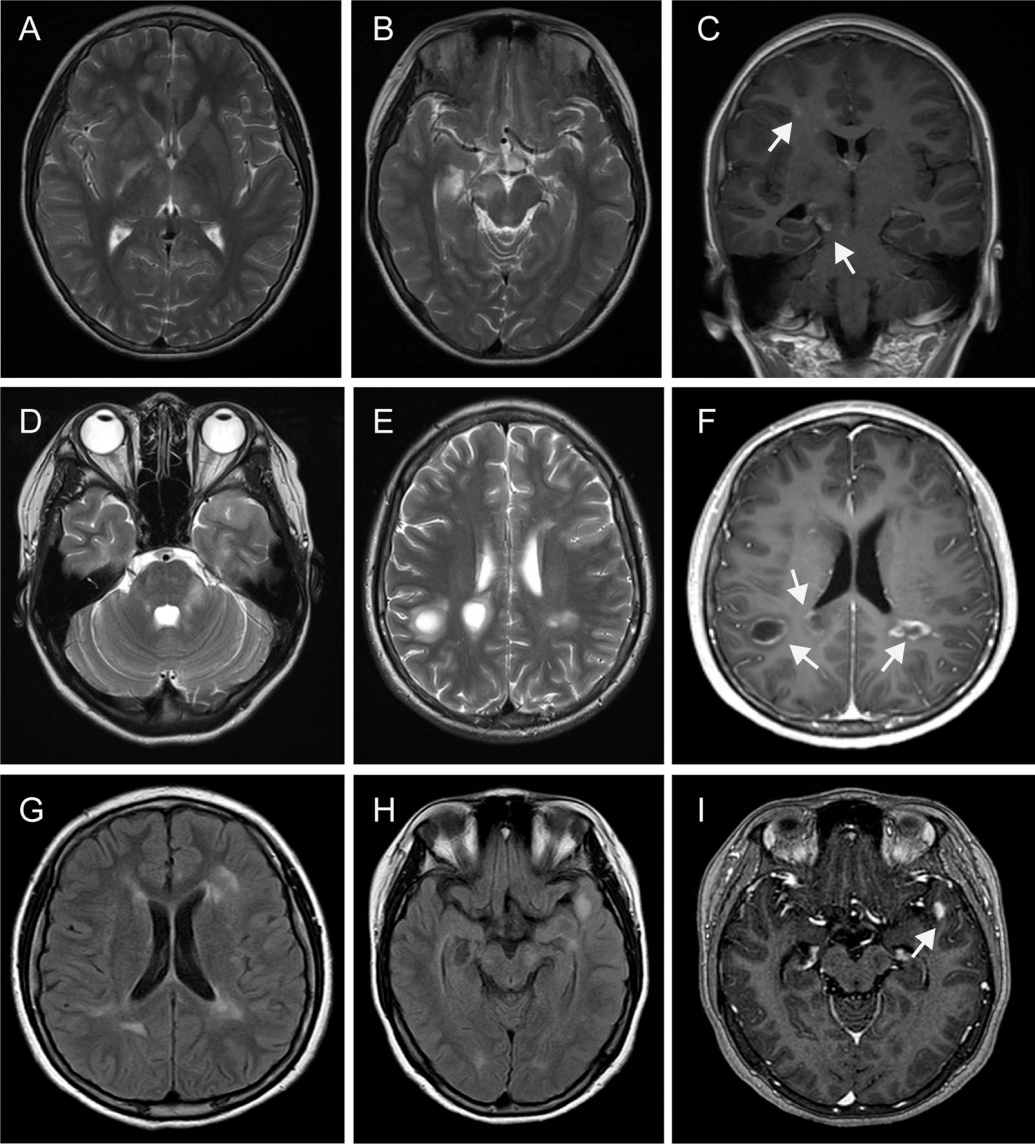
Magnetic resonance imaging findings. MRI performed in July 2012 demonstrated multiple T2 hyperintense lesions (periventricular, juxtacortical, thalamus, basal ganglia, caudex cerebri) **(A**, **B)**, some of them obtained with gadolinium enhancement [**(C)**, arrowhead]. Repeated MRI in Jan 2020 revealed multiple T2 hyperintense lesions (juxtacortical, periventricular, left brachium pontis), with some lesions appearing as “fried egg sign” **(D**, **E)**. Some of them with enhancement lesions appeared as “open sing sign” [**(F)**, arrowhead]. MRI in May 2020 exhibited multiple FLAIR hyperintense lesions (periventricular, juxtacortical, left caudex cerebri, left temporal lobe) **(G**, **H)**, with a left temporal lobe lesion showing gadolinium enhancement [**(I)**, arrowhead]. MRI, Magnetic resonance imaging; FLAIR, fluid attenuated inversion recovery.

**Figure 1** provides a graphical presentation of the case. A Chinese female presented with vision loss in the right eye and left limb paralysis in June 2012 at the age of 16. **Figures 2A–C** shows a brain magnetic resonance imaging (MRI) scan performed at that time. Brain MRI showed multiple T2-hyperintense lesions and some of the lesions obtained with gadolinium enhancement. One oligoclonal immunoglobulin G (IgG) band was found in the cerebrospinal fluid (CSF). She was diagnosed with MS and received immunoglobulin therapy resulting in complete recovery. One year later, the patient had another inflammatory demyelinating attack, with complaints of new-onset numbness of the left limb and vision loss in the right eye again in July 2013. She was administered with interferon β-1a for one year and maintained a clinically stable status until 2018. She experienced a spinal demyelinating episode in April 2018. Intravenous high-dose methylprednisolone pulse and intravenous immunoglobulin therapy were effective, she was discharged without neurological deficits and she remained free of relapses. According to the revised McDonald criteria (3), the diagnosis of RRMS was made.

At the age of 19, the patient was presented to our department on January 18, 2020 due to behavioral complaints, psychosis disorder, sleep dysfunction, and seizures that lasted for 15 days. She had also developed confusion, aggression, catatonia, memory deficit, and visual hallucinations within a few days. She exaggerated that she earned over 10,000 RMB monthly by live broadcast (family denied). She also presented with abnormal movements affecting the right limb and mandible. During the admission, the patient developed multiple episodes of psychomotor agitation and was unresponsive to a combination of neuroleptics and benzodiazepines. Neurological examination revealed intermittent and illogical speech, memory and cognition decreased significantly. Visual field testing showed no deficits. There were no cranial nerve abnormalities. Motor testing showed 5/5 strength in the upper and lower extremities, and deep tendon reflexes were normal in all limbs. The patient had no sensory deficits as well as pathological reflexes. Her score was 19/30 on the Montreal Cognitive Assessment scale, suggesting severe cognitive impairment. Brain MRI demonstrated multiple T2 hyperintense lesions (juxtacortical, periventricular, left brachium pontis), with some lesions appearing as “fried egg sign” (**Figures 2D, E**). Some brain MRI scans with enhancement lesions appeared as “open ring sign” (**Figure 2F**, arrowhead). Upon comparison of the MRI scans to those from eight years ago, an obvious increase of the lesion load was noted. Tests for metabolic encephalopathies were negative: complete blood cell count, erythrocyte sedimentation rate, C-reactive protein, folic acid, vitamin B12, liver and kidney function tests, antinuclear antibody, antithyroglobulin, antithyroperoxidase antibodies, ceruloplasmin, and laboratory tests for toxicology. Human immunodeficiency virus and rapid plasma regain were negative. CSF examination showed a mild lymphocytic pleocytosis (26 cells per uL) [normal range: 0–20], normal protein level (0.307 g/L) [normal range: 0.15–0.45], normal glucose level (4.92 mmol/L) [normal range: 3.80–6.1] and positive oligoclonal bands, which was not observed in corresponding serum. CSF Gram staining and culture yielded negative results. Immunological analyses using fixed cell-based assays were positive for specific anti-NMDAR antibodies in both the CSF (IgG against the NR1 subunit, 1:32) and serum (IgG against the NR1 subunit, 1:32). Tests for other autoimmune encephalitis and onconeural antibodies were negative both in the CSF and serum, including α-amino-3-hydroxy-5-methyl-4-isoxazolepropionic acid, mGluR5, γ-aminobutyric acid B, leucine-rich glioma-inactivated 1, contactin-associated protein-like 2, dipeptidyl-peptidase-like protein 6, glutamate decarboxylase 65, Hu, Yo, Ri, Ma2/Ta, and CV2/CRMP5. No informative autoantibodies were detected in the CSF and serum paraneoplastic evaluation. Tests for anti-aquaporin 4 and anti-myelin oligodendrocyte glycoprotein were revealed negative for both antibodies using fixed cell-based assays. A long-term video-electroencephalography showed high amplitude slow wave activities, bilateral forehead-frontal-temporal-occipital focus, and singular as well as grouped spike-wave complexes with abnormal rhythmizing. Various examinations, including antibodies for tumor markers, paraneoplastic syndromes, abdominal and pelvic computed tomography scans, and ultrasounds of the reproductive system, were performed to exclude neoplasm associated with anti-NMDA receptor encephalitis; each of these results was negative.

## Diagnostic Assessment

The patient was diagnosed with anti-NMDAR encephalitis overlapping with MS and administered with pulse therapy of immunoglobulin (IVIG, 0.4 g/kg) for 5 days, methylprednisolone (1 g/day) for 5 days (the dosage was decreased every 5 days), followed by oral prednisolone (30 mg/day), olanzapine (5 mg/day), and levetiracetam (1 g/day). After one month, of hospitalization the patient’s clinical status had significantly improved with the exception of abnormal movements and sleep disorder. She was discharged without neurological deficits, psycho-behavioral symptoms, or epileptic seizures 2 months later.

During the follow-up 4 months later, the patient complained of slight headache and vision loss of the right eye and was admitted to our hospital again. The findings from thorough neurological and psychiatric examinations were unremarkable, except for right visual loss. Her score was 30/30 on the Montreal Cognitive Assessment scale. Repeat CSF analysis revealed values within normal limits: white blood cells, 3 cells per uL [normal range: 0–20], primary mononuclear cells; total protein, 0.197 g/L [normal range: 0.15–0.45]; and glucose, 3.82 mmol/L [normal range: 3.80–6.1]). Upon testing for anti-NMDAR antibodies in the CSF using fixed cell-based assays, a 1:1 titer was revealed. Brain MRI showed contrast-enhancing lesions (**Figures 2G–I**) and she eventually started treatment in May 2020. Following a methylprednisolone pulse therapy complete remission was reached. The patient did not consider commencing disease-modifying treatment again due to economic reasons.

## Literature Review

A literature search was conducted using the PubMed database. The following combinations of search terms were used: “multiple sclerosis and anti-N-Methyl-D-Aspartate Receptor Encephalitis”, “multiple sclerosis and anti-NMDAR encephalitis”. The search was limited to articles in English. Available data in the form of abstracts or full-text articles and related citations and references were reviewed.

## Discussion

We describe a case diagnosed with RRMS who developed anti-NMDA-R encephalitis during the MS exacerbation phase. The clinical courses of the patient described in the report was compatible with that of MS, coupled with MRI evidence of dissemination in time and space, fulfilling both clinical and radiological criteria for RRMS (3). However, one of her relapsing MS courses was characterized by fulminant neuropsychiatric symptoms, including psychosis disorder, seizures, movement disorder, and sleep dysfunction, which are the “red flag” symptoms of anti-NMDA-R encephalitis (4) instead of MS. The occurrence of atypical symptoms of MS should raise suspicion of an autoimmune etiology and lead to inclusion of autoimmune encephalitis as another diagnosis. In our case, to investigate further for evidence, the anti-NMDAR antibodies were tested and the anti-GluR1 antibody highly specific for anti-NMDAR encephalitis was found to be positive, suggesting the diagnosis of anti-NMDAR encephalitis with overlapping MS. Anti-NMDA-R encephalitis cannot be ruled out when MS is combined with atypical symptoms (e.g., psychiatric symptoms, epilepsy, and movement disorder). Detection of antibodies related to autoimmune encephalitis is the key to efficient identification.

Besides, it is well-known that the differential diagnosis of multiple intracranial lesions is a challenging task. Although our case presented typical imaging findings of MS (e.g., “fried egg sign,” “open ring sign”), these features are neither specific nor unique. Moreover, NMDAR encephalitis can also present radiologically with multiple sclerosis-like demyelinating lesions (5). While an MS relapse may have been suspected, the clinical findings were largely consistent with NMDAR encephalitis. Therefore, it is not certain whether the acute MS relapse was accompanied by NMDA encephalitis.

The coexistence between anti-NMDAR encephalitis and MS had been reported in a few cases. The results of the literature review and the present case are shown in **Table 1**. Among the eight patients studied, two were males and six females. The average age was 31.3 years. The first case was reported from Japan (7), along with two from Germany (8, 9), three from England (6, 10) and one from Turkey (11). Our patient was the first case from China. In two patients, the symptoms of anti-NMDAR encephalitis preceded those of MS, while in six patients the trajectory opposite. In our case, anti-NMDAR encephalitis and MS exacerbation were observed simultaneously. All brain MRI findings were consistent with MS. Interestingly, no patient developed tumors and five of them showed good clinical prognoses. Two more cases were reported, one in a retrospective case-control study (only one patient with RRMS was found in 89 patients with demyelinating disorders) (12) and another in a review (only one patient was diagnosed with MS among 691 patients diagnosed with anti-NMDAR encephalitis) (13). In general, overlapping anti-NMDAR encephalitis with MS was found to be rare.

**Table 1:** Characteristics of patients with overlapping demyelinating disorders-NMDA encephalitis.

Course	Country	Author	Age/sex	Onset	Tumor	NMDAR Ab	Other immunology findings	MRI findings	Treatment	Prognosis
NMDAR->MS	England	Baheerathan et al. (6)	32 years/F	Encephalopathy, seizures, abnormal movements	No	In sera (+)	OB (+), AQP4 (-), MOG (-)	Consistent with demyelination	Not tolerated immunotherapy, improved spontaneously	Persisting cognitive impairment
NMDAR->MS	England	Baheerathan et al. (6)	29 years/M	Psychosis, seizures	No	In sera (+) In CSF (+)	OB (+)	Consistent with demyelination	None, improved spontaneously	Good
MS->NMDAR	Japan	Uzawa et al. (7)	33 years/F	Fatigue, seizures, lost consciousness, Psychosis	No	In sera (-) In CSF (+)	AQP4 (-)	Consistent with MS	IVMP	Good
MS->NMDAR	Germany	Waschbisch et al. (8)	33 years/M	Paroxysmal tingling, tonic spasms	No	In sera (+)	OB (+), AQP4 (-)	Consistent with MS	Rituximab	Good
MS->NMDAR	Germany	Fleischmann et al. (9)	33 years/F	Memory lost, cognitive deterioration, delusions	No	In sera (-) In CSF (+)	OB (+)	Consistent with MS and atrophy	Plasmapheresis, corticosteroids, mitoxantrone, IVMP	Bad
MS->NMDAR	England	Suleman et al. (10)	41 years/F	persecutory delusions, visual hallucinations, odd behaviour	No	In sera (+)	OB (+)	Unremarkable	Plasmapheresis, Rituximab, IVIG	Problems with short-term memory
MS->NMDAR	Istanbul	Gulec et al. (11)	26 years/F	Confusion, agitation, hallucinations	No	In sera (+)	OB (+), AQP4 (-), MOG (-)	Consistent with MS	Plasmapheresis, Rituximab, IVMP, IVIG	Good
MS->NMDAR	China	Our case	19 years/F	Psychosis, abnormal movement, seizures	No	In sera (+) In CSF (+)	OB (+), AQP4 (-), MOG (-)	Consistent with MS	IVMP, IVIG	Good

OB, oligoclonal immunoglobulin bands; MS, multiple sclerosis; RRMS, relapsing remitting multiple sclerosis; NMDAR, Anti-N-methyl-D-aspartate receptor encephalitis; AQP4, Anti-aquaporin 4 antibody; MOG, Anti-myelin oligodendrocyte glycoprotein antibody; M, male; F, female; IVMP, intravenous methylprednisolone; IVIG, intravenous immunoglobulin; CSF, cerebrospinal fluid.

Is the overlap of anti-NMDAR encephalitis and MS incidental or related? There may be a possible link between these diseases, and concurrent autoimmune responses may be important for the development of anti-NMDAR encephalitis (2). To our knowledge, two confirmed triggers of anti-NMDAR encephalitis are tumor (mostly ovarian teratomas) (1, 14) and viral herpes encephalitis (mostly simplex herpes encephalitis) (15). Another possible hypothesis is that inflammatory demyelination may be a trigger for approximately 5% of patients with anti-NMDAR encephalitis with clinical or radiological evidence of a demyelinating disorder, particularly with neuromyelitis optical spectrum disorder and myelin oligodendrocyte glycoprotein-associated disorders (2, 14). These studies suggest associations between the two autoimmune disorders that have clinical implications. Patients with an overlap between these demyelinating disorders often have a history of episodes of encephalitis or demyelinating syndromes and a relatively good prognosis (16). These patients are less likely to have an underlying tumor (16), suggesting that patients with non-paraneoplastic forms are prone to autoimmune disorders (2). The mechanisms connecting anti-NMDAR encephalitis with demyelinating disorders remain unclear. Both molecular mimicry and the breakdown of immunologic tolerance toward NMDARs released following neuronal damage have been described as possible mechanisms (8, 17, 18). MS is a demyelinating disorder, although its association with NMDA is rare, it still considered to be a related factor. The mechanism by which MS might give rise to anti-NMDAR encephalitis is uncertain, but the interaction between antigens of the central nervous system and intrathecal immune response has been conjectured (17, 18), which is that MS might expose antigens of the neurons and activate the intrathecal immune response concurrently. In our case, the patient did not receive sustained immunomodulatory therapy, which might have led to an exposure to neuronal antigens with possible implications for the recurrent demyelinating activity and anti-NMDA encephalitis.

Also, another hypothesis is that the use of multiple immunosuppressive therapies in MS may be related with immune-dysregulation and associated with autoimmune encephalitis in selective individuals (11). In a previous reported case, a retrospective diagnosis of anti-NMDAR encephalitis was made six years after a female patient diagnosed with RRMS had presented with severe cognitive impairment (9). The patient had a fulminant relapse and an increase of the anti-NMDAR IgG titer in the CSF after cessation of natalizumab administration. CD138+ plasma cell levels in the CSF of natalizumab-treated patients with MS are significantly lower compared with those of patients with other neurological diseases and untreated patients with MS but increase after discontinuation of natalizumab (19). Therefore, it seems plausible that natalizumab withdrawal facilitates entry of NMDAR antibody-producing plasma cells into the central nervous system (8). In our case, this possibility did not have to be considered since her overall immunotherapy was rather minimal with no ongoing immunomodulatory therapy.

## Conclusion

We describe a Chinese female patient diagnosed with relapsing remitting multiple sclerosis who developed anti-NMDAR encephalitis. Anti-NMDAR encephalitis should be considered when MS patients present atypical symptoms (e.g., psychiatric symptoms, epilepsy, and movement disorder), and testing for anti-NMDAR antibodies is needed in such situations. Our observations suggest that there might be a possible link between anti-NMDAR encephalitis and MS. Recognition of these associations is important to avoid misdiagnoses and refine the treatment.

## Data Availability Statement

The original contributions presented in the study are included in the article/supplementary material. Further inquiries can be directed to the corresponding authors.

## Ethics Statement

The studies involving human participants were reviewed and approved by Medical Ethics Committee of Shenzhen People’s Hospital. Shenzhen People’s Hospital. The patients/participants provided their written informed consent to participate in this study. Written informed consent was obtained from the individual(s) for the publication of any potentially identifiable images or data included in this article.

## Author Contributions

YH wrote the manuscript and was involved in the diagnostic and therapeutic clinical progress. QW, SZ, and YZ were involved in care of the patients. LZ supported the interpretation. XF revised the manuscript for intellectual content and was responsible for diagnostics and treatment of the patient. QX contributed to the figures and table and helped in the diagnostic process and critically revising the manuscript. All authors contributed to the article and approved the submitted version.

## Conflict of Interest

The authors declare that the research was conducted in the absence of any commercial or financial relationships that could be construed as a potential conflict of interest.

## Abbreviations

NMDAR, N-methyl-D-aspartate receptor; MS, multiple sclerosis; RRMS, relapsing remitting multiple sclerosis; MRI, magnetic resonance imaging; CSF, cerebrospinal fluid; IgG, Immunoglobulin G.
